# Supplementary material for: Loxin Reduced the Inflammatory Response in the Liver and the Aortic Fatty Streak Formation in Mice Fed with a High-Fat Diet
Source: Int J Mol Sci. 2022 Jun 30;23(13):7329. doi: 10.3390/ijms23137329 (PMC9266330; doi:10.3390/ijms23137329)
Supplement: Supplementary file 1 [file ijms-23-07329-s001.zip › ijms-1705833-supplementary.pdf]

Supplementary materials:

Table S1. Biochemical characteristics from ND and HFD animals.

	**ND**	**NFD**	**ND**	**NFD**
	Ado-null		Ado-LOXIN	
Glucose (mg/dL)	110±9.0	162±9*	100±1.0	152±9*
TC (mg/dL)	125±10	160±12*	115±1.0	174±8*
TG (mg/dL)	97±2.9	100±5	99±2.0	95±5
HDL-C (mg/dL)	20±3.0	21±4	19±2.0	18±3
ALT (UI)	51±2.0	91±3*	60±6.0	75±5**
AST (UI)	45±1.0	86±1*	50±2.0	75±5**

Data are expressed as mean± SEM. TC, total cholesterol; TG, triglycerides; HDL, high-density lipoprotein; ALT, alanine aminotransferase; AST, aspartate aminotransferase; * p < 0,05 vs ND. ** p < 0,02 vs HFD Ado-null.
